# Hair dye use, regular exercise, and the risk and prognosis of prostate cancer: multicenter case–control and case-only studies

**DOI:** 10.1186/s12885-016-2280-7

**Published:** 2016-03-21

**Authors:** Shu-Yu Tai, Hui-Min Hsieh, Shu-Pin Huang, Ming-Tsang Wu

**Affiliations:** Graduate Institute of Medicine, College of Medicine, Kaohsiung Medical University, Kaohsiung, Taiwan, R.O.C; Department of Family Medicine, School of Medicine, College of Medicine, Kaohsiung Medical University, Kaohsiung, Taiwan, R.O.C; Department of Family Medicine, Kaohsiung Municipal Ta-Tung Hospital, Kaohsiung, Taiwan, R.O.C; Department of Family Medicine, Kaohsiung Medical University Hospital, Kaohsiung Medical University, Kaohsiung, Taiwan, R.O.C; Department of Public Health, College of Health Sciences, Kaohsiung Medical University, Kaohsiung, Taiwan, R.O.C; Department of Urology, Kaohsiung Medical University Hospital, Kaohsiung Medical University, Kaohsiung, Taiwan, R.O.C; Department of Urology, Faculty of Medicine, College of Medicine, Kaohsiung Medical University, Kaohsiung, Taiwan, R.O.C; Center of Environmental and Occupational Medicine, Kaohsiung Municipal Hsiao-Kang Hospital, Kaohsiung Medical University, Kaohsiung, Taiwan, R.O.C

**Keywords:** Prostate cancer, Hair dye, Regular exercise, Risk, Survival, Case–control study

## Abstract

**Background:**

This study investigated the effects that hair dye use and regular exercise exert on the risk and prognosis of prostate cancer.

**Methods:**

We studied 296 cases of histologically confirmed prostate cancer and 296 age- (in 2-y bands), ethnicity-, and hospital-matched controls in Taiwan between August 2000 and December 2008. To determine the rate of prostate cancer survival, another 608 incident prostate cancer cases occurring between August 2000 and December 2007 were investigated. Information on hair dye use and regular exercise was obtained using a standardized questionnaire.

**Results:**

The use of hair dyes was associated with a significant 2.15-fold odds of developing prostate cancer (adjusted odds ratio = 2.15, 95 % confidence interval [CI] = 1.32–3.57), but was not associated with prostate cancer survival, compared with no use. The significant risks were more prominent in users aged < 60 years who had used hair dyes for > 10 years, > 6 times per year, and started using hair dyes before 1980. By contrast, regular exercise significantly reduced the number of prostate-cancer-specific death (adjusted hazard ratio = 0.37, 95 % CI = 0.16–0.83); the protective effect of exercise was more prominent among cancer patients who exercised daily (≥7 times/week). However, exercise could not prevent the development of prostate cancer.

**Conclusions:**

Hair dye use increased the risk of prostate cancer, whereas regular exercise reduced the number of prostate-cancer-specific deaths.

**Electronic supplementary material:**

The online version of this article (doi:10.1186/s12885-016-2280-7) contains supplementary material, which is available to authorized users.

## Background

Prostate cancer is the most frequently diagnosed cancer, second only to skin cancer, and the second-leading cause of cancer death in the United States; it is estimated to have caused 27,050 deaths in 2007 [1]. Although the incidence rate of prostate cancer was reported to be lower among Asian populations than among Caucasian populations, the annual incidence rate of prostate cancer in Taiwan had increased at least 2-fold from 5.7 per 10^5^ people in 1995 to 12.1 per 10^5^ people in 2007 [2]. Various etiologic studies have suggested that the strongest risk factors for prostate cancer comprise older age, a family history of the disease, and African American ethnicity [3, 4]; however, numerous genetic and environmental risk factors remain undetermined.

Accumulated evidence has indicated that 80 to 90 % of human cancers might be attributable to environmental and lifestyle factors such as dietary or cosmetic habits, physical activities, and substance use [5–7]. Among these factors, hair dye use and regular exercise are 2 common practices in daily life [8, 9].

Hair-coloring product sales are estimated to have a market of approximately US$12 billion per year worldwide, and as much as 50 % of the adult population in developed countries has used hair colorants [9]. Hair coloring products include a wide range of more than 5,000 chemical substances, some of which have been reported to be mutagenic and carcinogenic according to various bioassay results [10]. Numerous oxidative dyes were reformulated in the early 1980s to eliminate ingredients that induced tumors; however, whether current compounds exert carcinogenic effects or affect overall immune responses remains unclear [11, 12]. Relevant studies have reported scant evidence regarding the association between hair dye use and cancer risk, except for a possible cause of hematopoietic cancers [13–18] and bladder cancer [11, 15, 19–23]. No epidemiologic studies have investigated the relationship between hair dye use and the risk and prognosis of other genitourinary tract cancers such as prostate cancer.

Numerous studies have examined whether increased physical activity can reduce the risk of prostate cancer [24–30]; the conclusion remains conflicting, although most studies have reported no association between them [24, 26, 28–30]. Moreover, few studies have examined whether physical activity can improve the prognosis of prostate cancer. Thus, in this study, we attempted to clarify the relationships among hair dye use, regular exercise, and the risk and prognosis of prostate cancer. We hypothesized that increased hair dye use and decreased regular exercise would increase the risk of prostate cancer and affect the prognosis of patients with prostate cancer.

## Methods

### Study populations

To investigate the risk of prostate cancer, we conducted a hospital-based case–control study at 2 large medical centers: Kaohsiung Medical University Hospital (KMUH) and Kaohsiung Veterans General Hospital (KVGH), located in Southern Taiwan. Case patients comprised men who had been newly diagnosed with and pathologically proven to have adenocarcinoma of the prostate between August 2000 and December 2008. We matched each case patient with one healthy man (control) who received a health check-up in the Department of Preventive Medicine during the same month that the case patient was diagnosed; the patients and controls were frequency matched according to age (in 2-y bands), ethnicity, and hospital of origin. The controls had undergone digital rectal examinations, the results of which were normal, and had serum prostate-specific antigen (PSA) levels lower than 4 ng/dL.

To investigate the survival rate of patients with prostate cancer, we conducted a case-only study, recruiting patients newly diagnosed with adenocarcinoma of the prostate at the Third Medical Center at National Taiwan University Hospital (NTUH) in Northern Taiwan between August 2000 and December 2007. Because the National Death Registry of Taiwan has released the personal information, health status, and cause of death for patients diagnosed before December 2007, we studied only the cases of patients who were diagnosed before December 2007 in this case-only study. The 3 hospitals are the main medical centers in their geographic areas and are accessible to patients from all socioeconomic groups in Taiwan.

### Data collection

Participants in the case–control and case-only studies underwent in-person interviews conducted by trained interviewers using standardized questionnaires. The interviewers questioned the paired case patients and the controls regarding demographic and lifestyle characteristics before they were diagnosed with prostate cancer. The questionnaire included questions pertaining to the demographic characteristics of age, body height and weight (used to calculate body mass index [BMI]), education attainment, marital status, blood type, vasectomy history, and family history of cancer. In addition to the studied exposure factors (hair dye use and regular exercise), we collected other common and relevant environmental and lifestyle factors, such as diet and habitual substance use, including cigarette smoking, alcohol consumption, and betel nut chewing.

Cigarette smokers, alcohol drinkers, and betel nut chewers were separately defined as participants who had smoked 10 cigarettes per week for a minimum of 6 months; or consumed any alcoholic beverage once per week for a minimum of 6 months; or chewed one betel nut quid per day for a minimum of 6 months, respectively. The age at which substance use began and ceased, the type of substance, and the daily consumption amount and duration of use were documented for identified substance users [31]. The accuracy of information pertaining to substance use that was obtained from the questionnaires has been validated in our previous studies on esophageal cancer [32–34].

### Assessment of hair dye use and exercise status

Hair dye habit was defined as dyeing the hair a minimum of once every 3 months for at least 1 year. Detailed information regarding the age at first and final use, frequency, and years of use were recorded for identified hair dye users. Regular exercise habits were assessed by asking participants whether they exercised aerobically for a minimum of 20 min and perspired, performing this activity regularly for at least 1 year. If participants had regular exercise habits, we asked them to report their average exercise frequency according to 5 choices (≥1 time/d; 4–6 times/week; 1–3 times/week; 1–4 times/month; and < 1 time/month).

### Clinical characteristics

The clinical-pathological characteristics, including the Gleason score, stage of disease, and serum PSA level at diagnosis, were obtained from chart review and are described in detail elsewhere [35, 36]. Disease stage was determined by analyzing the pathological findings, pelvic computed tomography or magnetic resonance imaging, and radionucleotide bone scans, according to the criteria established by the American Joint Committee on Cancer (AJCC) tumor, node, and metastasis classification system (AJCC Cancer Staging Manual, Fifth Edition, 1997). The pathologic grade was determined according to Gleason scores and was classified into 3 groups (≤6, 7, or 8–10) [37]. Information on death from any cause was obtained from the National Death Registry of Taiwan, which is accurate and complete because death registration is mandatory in Taiwan and physicians must issue death certificates [38]. This study was approved by the Institutional Review Board of the Kaohsiung Medical University Chung-Ho Memorial Hospital, Kaohsiung Veterans General Hospital, and the Research Ethics Committee of National Taiwan University Hospital. The written informed consent was obtained from all the study participants of the 3 medical centers prior to participation.

### Statistical analysis

Demographic and clinical characteristics were tabulated for the cases and controls in the case–control study. Multivariable unconditional logistic regression models were used to estimate the odds ratios (ORs) and 95 % confidence intervals (CIs) for the relationships among hair dye use, regular exercise, and the risk of prostate cancer after adjustment for other covariates. Initially, we included the variables that have been considered significant risk factors or protective factors for prostate cancer in previous studies, including age (>65 y, ≤ 65 y), education attainment (< high school, high school, > high school), and family history of prostate cancer (yes, no). Missing data were classified into an additional category in the models to maximize the study participants. The additional variables were then added to the models according to forward stepwise selection, and were included in the models if they caused a minimal 10 % change in the association between hair dye use or regular exercise and prostate cancer risk for the risk of or protection against prostate cancer. The selected variables included marital status, BMI (<24 kg/m^2^, 24–26 kg/m^2^, ≥ 27 kg/m^2^), cigarette smoking (yes, no), alcohol consumption (yes, no), betel nut chewing (yes, no), blood type, vasectomy history, and food or nutrient intake (multivitamin supplements, tea, coffee, milk, soy products, and instant noodles). Because the intake frequency of specific nutritional supplements—including vitamins A, B, C, D, and E, and calcium—was less than 5 % among the study participants, we merged them into the multivitamin supplement category. The covariates used in the final model of the case–control study comprised age, marital status, blood type, education attainment, family history of prostate cancer, cigarette smoking, alcohol consumption, and betel nut chewing. We used an additional model, which only adjusted with age and family history of prostate cancer that is the risk factors for prostate cancer with sufficient evidence as race.

In addition to analyzing whether participants used hair dyes (yes, no), we categorized hair dye use based on age at first use (<50 y, 50–60 y, or ≥ 60 y), duration of use (≤10 y and > 10 y), frequency of use (<6 times/y and > 6 times/y), and year of first use (before and after 1980, which is the approximate year of the reformulation of dye products). We categorized exercise by frequency (1–6 times/week and ≥ 7 times/week).

For the case-only study, Kaplan–Meier analysis and log-rank testing were used to examine the relationship between personal hair dye use or regular exercise and the prostate cancer patient survival rate. Cox proportional hazards modeling was employed to compute hazard ratios (HRs) and 95 % CIs for prostate cancer deaths after adjustment for other covariates. The covariates included in the model were the clinical stage, PSA level, and the same covariates used in the case–control study. Each participant accumulated person-time beginning from the prostate cancer diagnostic date and ending on the date of prostate-cancer-related death or the end of this study in December 2007. If patients died from other causes, they were censored to account for the competing death attributable to other causes [39]. In addition, we analyzed the effect that exposure (hair dye use or regular exercise) exerted on the death attributable to other causes by censoring the study participants with prostate-cancer-specific deaths. Data analysis was performed using the SAS 9.1 statistical package; all *P* values were 2-sided and significant below the .05 level.

### Sensitivity analysis

Sensitivity analyses were conducted in both the case–control study and case-only study. In the matched case–control study, we excluded the case patients with a hair dye exposure history of less than 5 years before the occurrence of prostate cancer to account for a latent period, and examined the robustness of ORs for hair dye use. Because we did not inquire about the beginning or cessation of regular exercise among the study participants, we could not analyze how this variable affected the risk of prostate cancer. For the case-only study, we analyzed the case patients who were recruited before December 2006. For the missing data, in addition to treating them as an additional category, we also analyzed the participants without missing data [40].

## Results

**Fig. 1:**
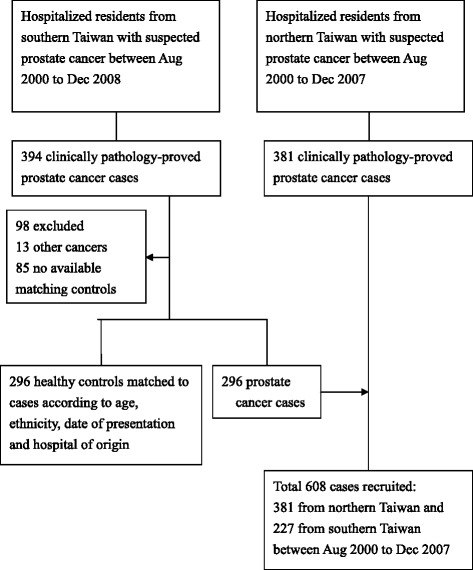
Study flowchart

Between August 2000 and December 2008, 394 participants, aged 50 to 90 years, were pathologically diagnosed with prostate cancer. After excluding cases in which patients were diagnosed with other cancers (*N* = 13) or that had no available matching control (*N* = 85), we obtained 296 cases and 296 controls for the final statistical analysis (Fig. 1). The numbers of patients who dyed their hair and exercised were 95 (32.1 %) and 174 (58.8 %) in 296 cancer patients, and 28 (28.6 %) and 49 (50.0 %) in 98 excluded cancer patients. No significant differences in exposure distributions were observed between included and excluded cancer patients (χ^2^ = 0.426 and 2.313; *P* = .514 and .128). For the survival analysis, 608 pathologically diagnosed prostate cancer patients (227 from KMUH and KVGH, and 381 from NTUH) were analyzed.

Table 1 lists the main demographic and clinical characteristics for the case–control and case-only studies (Additional file 1: Table S1 for other variables). In the case–control study, the average age and BMI of the study population were 71.6 years and 24.3 kg/m^2^, respectively. Except for the variables of marital status and blood type, the frequency of other variables was non-significant between cases and controls (Table 1 and Additional file 1: Table S1).

**Table 1:** Demographic and clinical characteristics of the 296 matched case–control study and the 608 case-only study

	Cases		Controls			All cases		
Variables	*N* = 296		*N* = 296		*P*-value	*N* = 608		
	N	(%)	N	(%)		N	(%)	Death^a^ (*N* = 48)
Study site1								
South	296	(100.0)	296	(100.0)		227	(37.3)	19
North	-		-			381	(62.7)	29
Age (yrs)								
Mean ± SD	71.3 ± 7.4		70.9 ± 8.0		0.622	71.6 ± 8.6		
<65	58	(19.6)	64	(21.6)	0.430	133	(21.9)	8
≥65	229	(77.4)	215	(72.6)		471	(77.5)	40
Missing	9		17			4		
BMI (kg/m2)								
Mean ± SD	24.2 ± 3.1	24.1 ± 3.2	0.742	24.3 ± 3.2				
<24	130	(43.9)	134	(45.3)	0.967	277	(45.6)	27
24– < 27	90	(30.4)	89	(30.1)		207	(34.0)	13
≥27	41	(13.9)	43	(14.5)		98	(16.1)	5
Missing	35		30			26		3
Education								
<high school	100	(33.8)	111	(37.5)	0.861	175	(27.8)	18
High school	85	(28.7)	86	(29.1)		225	(37.0)	17
>high school	66	(22.3)	66	(22.3)		178	(29.3)	13
Missing	45		33			30		0
Marital status*								
Single/widowed/separated/divorced	44	(14.9)	30	(10.1)	0.047*	55	(9.0)	6
Married	205	(69.3)	231	(78.0)		520	(85.5)	42
Missing	47		35			33		
Vasectomy								
No	215	(72.6)	199	(67.2)	0.628	516	(84.9)	39
Yes	10	(3.4)	7	(2.4)		17	(2.8)	0
Missing	71		90			75		9
Family history of PC								
No	226	(76.4)	245	(82.8)	0.243	523	(86.0)	43
Yes	17	(5.7)	11	(3.7)		32	(5.3)	3
Missing	53		40			53		
Cigarette Smoking								
No	153	(51.7)	159	(53.7)	0.561	293	(48.2)	20
Yes	143	(48.3)	135	(45.6)		315	(51.8)	28
Missing	0		2			0		0
Alcohol drinking								
No	199	(67.2)	208	(70.2)	0.459	428	(70.4)	28
Yes	95	(32.1)	87	(29.4)		179	(29.4)	20
Missing	2		1			1		0
Betel nut chewing								
No	281	(94.9)	281	(94.9)	0.845	578	(95.1)	44
Yes	13	(4.4)	12	(4.1)		28	(4.6)	3
Missing	2		3			2		1
Clinical stage^b^								
Localized						290	(47.7)	7
Locally advanced						158	(26.0)	7
Bone metastasis						154	(25.3)	34
Missing						6		0
Gleason score								
<6						255	(41.9)	11
7						191	(31.4)	12
8-10						154	(25.3)	25
Missing						8		0
Preoperative PSA (ng/ml)								
<10						152	(25.0)	5
10- < 20						143	(23.5)	4
≥20						295	(48.5)	38
Missing						18		1

*Abbreviation*: *BMI* body mass index, *PC*: prostate cancer, *PSA* prostate-specific antigen

1*Study site*: *North* National Taiwan University Hospital, *South*: Kaohsiung Medical University Hospital and Kaohsiung Veterans General Hospital

*P-value < 0.05; ^a^prostate cancer specific death; ^b^Tumor, node, metastasis system staging by American Joint Committee on Cancer (1997): Localized, T1/T2 N0 M0; Locally advanced, T3/T4 N1 M0; Bone Metastasis, M1

The use of hair dye was more prevalent among case patients than controls (32.1 % vs 21.6 %; Table 2). After adjustment for other covariates, the development of prostate cancer in hair dye users was 2.15-fold higher (95 % CI = 1.32–3.57) than that in nonusers. Among the users, the average (± SD) number of years of hair dye use was 13.9 ± 10.9, ranging from 1 to 50 years. The significant risks were more prominent in the users aged < 60 years who had used hair dyes for > 10 years and > 6 times per year, and who had started using them before 1980. By contrast, no protective effect was observed for participants who exercised regularly (Table 2). Although we restricted our analyses to participants without missing data, the results remained similar (Additional file 1: Table S2).

**Table 2:** Odds ratio (OR) for cases and controls according to hair dyes use and regular exercise

	Cases		Controls		Crude OR (95 % CI)	AOR (95 % CI)^a^	AOR (95 % CI)^b^
Variables	*N* = 296		*N* = 296				
	N	(%)	N	(%)			
Hair dyes							
No	197	(66.6)	231	(78.0)	1.00	1.00	1.00
Yes	95	(32.1)	64	(21.6)	1.74 (1.21–2.53)*	1.86 (1.21–2.82)*	2.15 (1.32–3.57)*
Missing	4		1		4.69 (0.69–92.10)	5.03 (0.73–98.83)	5.40 (0.76–108.02)
Age of first use (yrs)							
Never	197	(66.6)	231	(78.0)	1.00	1.00	1.00
≥60	24	(8.1)	21	(7.1)	1.34 (0.72–2.49)	1.23 (0.63–2.39)	1.52 (0.71–3.30)
50- < 60	28	(9.5)	21	(7.1)	1.56 (0.86–2.87)*	2.19 (1.08–4.60)*	2.64 (1.16–6.36)*
<50	29	(9.8)	16	(5.4)	2.13 (1.14–4.11)*	2.32 (1.21–4.61)*	2.25 (1.01–5.19)*
Missing	18		7		3.02 (1.28–7.89)	2.70 (1.04–7.86)	4.29 (1.32–16.72)
P for trend					<0.01	<0.01	<0.01
Years of use (years)							
Never	197	(66.6)	231	(78.0)	1.00	1.00	1.00
≤10	40	(13.5)	35	(11.8)	1.34 (0.82–2.20)	1.49 (0.87–2.59)	1.57 (0.82–3.03)
>10	37	(12.5)	22	(7.4)	1.97 (1.13–3.50)*	2.10 (1.15–3.92)*	2.54 (1.23–5.41)*
Missing	22		8		3.22 (1.46–7.87)	3.23 (1.36–8.52)	5.43 (1.82–20.13)
P for trend					0.02	<0.01	<0.01
Frequency of use (times per year)							
Never	197	(66.6)	231	(78.0)	1.00	1.00	1.00
≤6	41	(13.9)	32	(10.8)	1.50 (0.91–2.49)	1.64 (0.95–2.85)	1.73 (0.91–3.32)
>6	35	(11.8)	25	(8.4)	1.64 (0.95–2.86)	1.97 (1.07–3.69)*	2.65 (1.26–5.78)*
Missing	23		8		3.37 (1.53–8.19)	2.82 (1.23–7.08)	3.85 (1.41–11.74)
P for trend					0.02	<0.01	<0.01
Year of first use							
No	197	(66.6)	231	(78.0)	1.00	1.00	1.00
After 1980	5	(1.7)	3	(1.0)	1.95 (0.47–9.62)	1.25 (0.23–6.83)	0.89 (0.11–5.90)
Before 1980	76	(25.7	55	(18.6)	1.62 (1.09–2.41)*	1.87 (1.21–2.90)*	2.16 (1.28–3.68)*
Missing	18		7		3.02 (1.28–7.89)	2.71 (1.04–7.89)	4.32 (1.33–16.87)
Regular exercise							
No	126	(42.6)	120	(40.5)	1.00	1.00	1.00
Yes	166	(56.1)	168	(56.8)	0.93 (0.67–1.28)	0.99 (0.69–1.43)	1.01 (0.64–1.59)
Missing	4		8		0.48 (0.02–5.03)	none	1.03 (0.66–1.62)
Frequency of exercise (times per week)							
No	126	(42.6)	120	(40.5)	1.00	1.00	1.00
1-6	56	(18.9)	65	(22.0)	1.22 (0.79–1.89)	0.94 (0.58–1.53)	1.01 (0.55–1.86)
≥7	110	(37.2)	103	(34.8)	0.98 (0.68–1.42)	1.02 (0.68–1.54)	0.97 (0.58–1.62)
Missing	4		8		0.48 (0.12–1.55)	1.57 (0.25–12.15)	0.97 (0.11–8.50)

*Abbreviation*: AOR: adjusted OR; OR: odds ratio; PC: prostate cancer

^a^Adjusting for age, and family history of PC, ^b^Adjusting for age, marital status, blood type, education, family history of PC, cigarette smoking, alcohol drinking and betel nut chewing

*P-value < 0.05

Among the 608 pathologically proven prostate cancer patients, 99 died during the study period; among them, 48 (48.5 %) died from prostate cancer and 51 (51.5 %) died from other diseases (Table 1). The mean and median follow-up times were 25.7 and 22.2 months, respectively, (range, 0.1–84.4 months). The use of hair dye was not correlated with the clinical stage of prostate cancer (categorized by localized, locally advanced, and bone metastasis), whereas regular exercise was highly correlated with the clinical stage (Additional file 1: Table S3). In addition, the Gleason score and preoperative PSA were significantly and positively associated with the clinical stage after demographic characteristics were adjusted for (Additional file 1: Table S3).

**Fig. 2:**
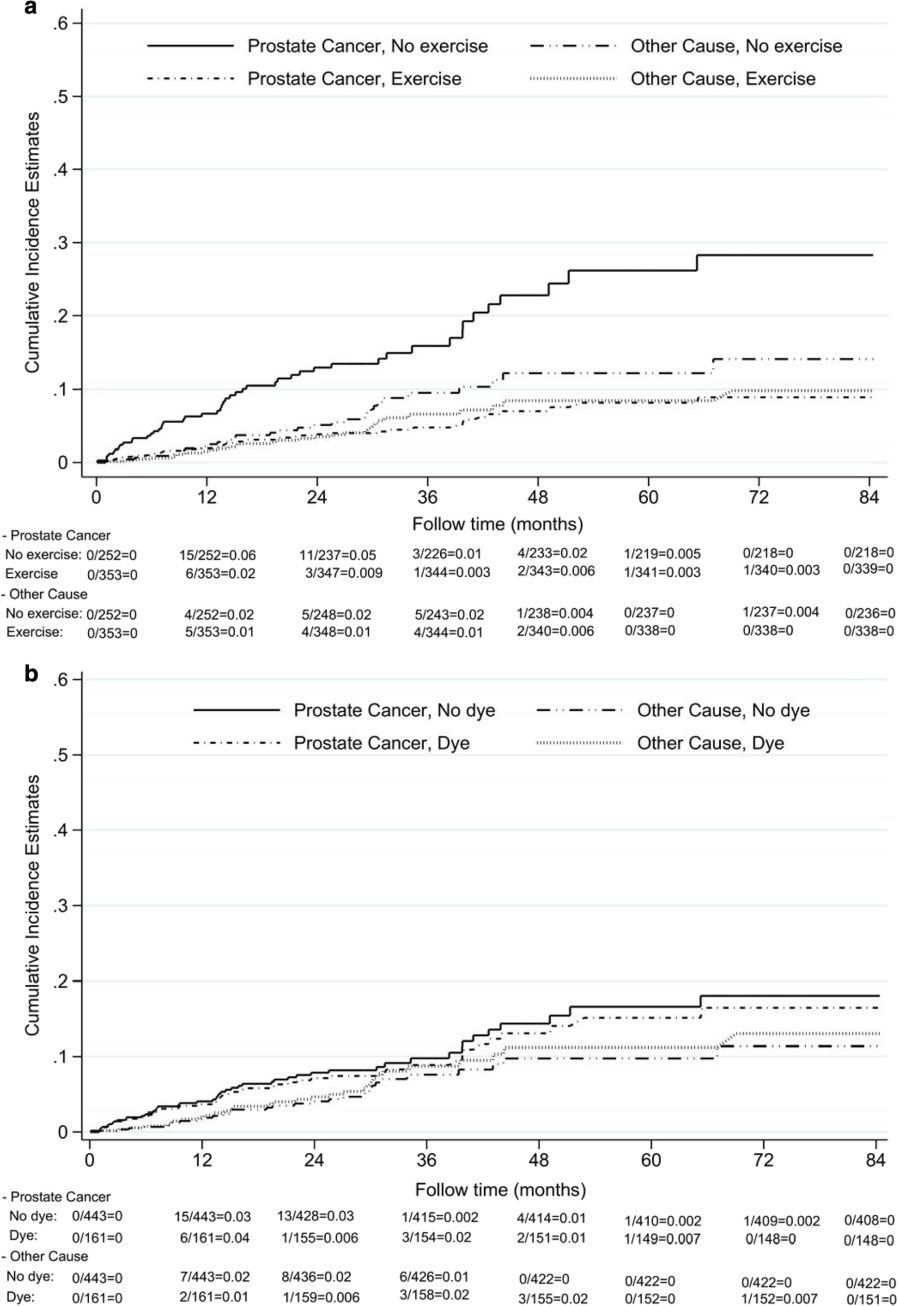
Cumulative incidence estimates of prostate-cancer specific death and other-cause death categorized by regular exercise or hair dye use: **a** by regular exercise; **b** by hair dye use

Cumulative incidence estimates of prostate-cancer-specific deaths were statistically dissimilar between patients who exercised and those who did not (*P* < .001; Fig. 2a). No significant relationship was present among other causes of death (*P* = .278). The use of hair dye did not affect cumulative incidence estimates of prostate-cancer-specific deaths or deaths from other causes (*P* = .753 and .693; Fig. 2b).

Regular exercise caused a 63 % decrease in the risk of prostate-cancer-specific death (adjusted *HR* = 0.37; 95 % *CI* = 0.16–0.83) after other covariates were adjusted for (Table 3). A significantly protective effect was prominent among cancer patients who exercised daily (≥7 times/week). By contrast, no significance was observed for the use of hair dye (Table 3). As expected, bone metastasis was the most crucial predictor of prostate-cancer-specific death (Additional file 1: Figure S1; Table 3). To avoid “reverse causality,” we dichotomized the cancer patients into 2 groups based on the absence or presence of bone metastasis. We found that the protective effect of regular exercise on the prognosis of prostate-cancer-specific deaths was significant in the group of bone metastasis patients, but not in the group of non-bone metastasis patients (Additional file 1: Tables S4 and S5).

**Table 3:** Prostate-cancer-specific death according to hair dye use and regular exercise in a Cox regression model

N	Total 608		Prostate-cancer-specific death 48	HR	(95 % CI)	AHR^a^	(95 % CI)
Variables	N (%)		N				
Hair dyes							
No	443	(72.9)	35	1.00		1.00	
Yes	161	(54.4)	13	0.89	(0.45-1.65)	1.14	(0.47-2.59)
Missing	4		0				
Years of use							
Never	443	(72.9)	35	1.00		1.00	
≤10 years	85	(14.0)	9	1.16	(0.52-2.31)	1.46	(0.54-3.56)
>10 years	64	(10.5)	2	0.35	(0.06-1.15)	0.33	(0.02-1.74)
Missing	16		2				
Frequency of use (times per year							
Never	443	(72.9)	35	1.00		1.00	
≤6	83	(13.7)	5	0.75	(0.26-1.74)	0.62	(0.14-1.94)
>6	63	(10.4)	7	1.07	(0.43-2.28)	2.15	(0.70-6.03)
Missing	19		1				
Regular exercise							
No	252	(41.4)	34	1.00		1.00	
Yes	353	(58.1)	14	0.27	(0.14-0.50)*	0.37	(0.16-0.83)*
Missing	3		0				
Frequency of exercise (times per week)							
Never	252	(41.4)	34	1.00		1.00	
1-6	161	(26.5)	7	0.34	(0.14-0.73)*	0.66	(0.22-1.82)
≥7	188	(30.9)	6	0.20	(0.08-0.45)*	0.23	(0.07-0.65)*
Missing	7		1				
Clinical stage^b^							
Localized	290	(47.7)	7	1.00		1.00	
Locally advanced	158	(26.0)	7	1.95	(0.67-5.71)	1.56	(0.41-5.69)
Bone metastasis	154	(25.3)	34	12.10	(5.64-29.99)*	10.15	(3.22-35.98*
Missing	6		0				

*Abbreviation*: *AHR* adjusted HR, *HR*: hazard ratio

*P-value < 0.05; ^a^Adjusting for clinical stage, PSA, age, marital status, blood type, education, family history of PC, cigarette smoking, alcohol drinking, and betel nut chewing

^b^Tumor, node, metastasis system staging by American Joint Committee on Cancer (1997): Localized, T1/T2 N0 M0; Locally advanced, T3/T4 N1 M0; Bone Metastasis, M1

Regarding deaths from other causes, regular exercise exerted a protective but non-significant effect (adjusted *HR* = 0.52; 95 % *CI* = 0.19–1.39; Additional file 1: Table S4). Neither hair dye use nor the clinical stage was significantly associated with deaths from other causes after other covariates were adjusted for (Additional file 1: Table S6).

Regarding the sensitivity analysis performed in the case–control study, because all participants were diagnosed with prostate cancer after at least 5 years of hair dye exposure, the results were unchanged. In the case-only study, we restricted the analysis to case patients (*N* = 445) who were recruited before December 2006, thus determining that the results were similar (Additional file 1: Table S7).

## Discussion

This study found an association that persisted after confounding adjustment, indicating that the use of hair dye may increase the risk of prostate cancer, but was unrelated to the clinical stage and prostate-cancer-specific death. The increased risk was observed in study patients who started to use hair dye products before 1980. Dose–response effects of increased exposure duration and frequency were also observed.

Few observational studies have investigated the association between hair dye use and prostate cancer risk, and their findings have been inconsistent [21, 41]. Guberan et al. examined 703 male and 677 female registered hairdressers who began managing salons in Geneva, Switzerland, between 1900 and 1964. The study participants were followed up until the end of 1982, and any incident of cancer between 1970 and 1980 was recorded [21]. The author observed an increased incident risk of all neoplasms, including prostate cancer, among the men (observed = 12 incident cases, expected = 6.1 incident cases; *P* < .05). Because the personal information in their study included only sex, age, marital status, and occupation, the confounding bias caused by other lifestyle factors was unavoidable. Another ecological study on cancer mortality among professions that involve making contact with hair dyes in the United States determined that no increased prostate cancer mortality rates existed [41].

Hair dyes manufactured before the 1980s contained carcinogenic agents such as aromatic amines and *p*-phenylenediamine that can be absorbed through the skin and are suspected to be among the main causes of urothelial cancer in humans [42–45]. Thus, in 1979, the U.S. Food and Drug Administration required a cancer warning label to be placed on the packaging of various hair dye products [46], and from 1978 to 1982, all oxidative dye products were reformulated to eliminate ingredients that were reportedly mutagenic or carcinogenic [16, 46]. The year in which dye formulas were changed is consistent with our results that a first use of hair dye before 1980 increased the risk of prostate cancer. Although the exact effect that the carcinogenic agents in hair dyes exert on the prostate is unknown, the carcinogens could possibly be absorbed through the urothelial epithelium and accumulate in the prostate gland, contributing to the malignancy of the prostate.

Regular exercise significantly reduces prostate-cancer-specific death, particularly in patients with bone metastasis. By contrast, regular exercise may not protect against developing prostate cancer, a result that was verified in the sensitivity analysis. Although numerous epidemiologic studies have examined the relationship between physical activity and the risk of prostate cancer [26–28, 30, 47–55], few of them have explored its effect on prostate-cancer-specific mortality. Currently, approximately 40 human studies have investigated physical activity and the risk of prostate cancer; however, less than 40 % of these studies (14 studies) have determined a significant protective effect. Most recent human studies have focused on the effect that regular exercise exerts on clinically advanced prostate cancer or prostate-cancer-specific death [29, 48, 49, 53–55], and most of them have suggested a protective effect with higher levels of recreational physical activity [48, 49, 53, 54]. However, they have observed no association between recreational physical activity and overall prostate cancer risk [48, 53].

The protective mechanism of physical activity on the survival of prostate cancer patients is likely attributable to the influence of various hormones, such as insulin [56, 57] and androgens [57], and other growth factors that are related to the aggressiveness of prostate cancer. Physical activity provides another protective mechanism that enhances immune function and antioxidant defense mechanisms, resulting in the increase of the survival rate of prostate cancer patients.

A limitation of this study was that the controls comprised volunteers who participated in health check-ups; thus, this group might not have been representative of the general population. Another limitation is that this was a multicenter hospital-based case–control study, and the exposures of interest were determined using a questionnaire; therefore, recall bias was likely. Although we verified the accuracy of various questionnaire items, such as cigarette smoking, alcohol consumption, and betel nut chewing obtained from previous studies [32–34], the bias was unavoidable. In addition, other unmeasured confounders might have been present. Another limitation is that no detailed information regarding the various brands of or ingredients in the hair dyes, which might have contained various levels of carcinogenic chemicals, was included. However, we asked “What year did you start using hair dyes, before or after 1980?”. Although we did not ask about the color of hair dyes, in Taiwan, most people use black or dark hair dyes to dye their grey hair and appear younger. Finally, we obtained no information on regular exercise performed after diagnoses of prostate cancer. Therefore, we were unable to clarify whether the protective effect of regular exercise on the survival of prostate cancer patients was caused by the benefit of exercise before or after the diagnosis, or whether the 2 were highly correlated.

## Conclusions

In conclusion, we determined that hair dye use was associated with an increased prostate cancer risk; however, hair dye use was unrelated to the survival of prostate cancer patients. By contrast, regular exercise reduced the risk of prostate-cancer-specific death but did not protect against the risk of prostate cancer.

## Additional file

Additional file 1: The supplementary tables and figures. (DOC 418 kb)

## Abbreviations

AJCC: American Joint Committee on Cancer
BMI: body mass index
CIs: confidence intervals
HRs: hazard ratios
KMUH: Kaohsiung Medical University Hospital
KVGH: Kaohsiung Veterans General Hospital
NTUH: National Taiwan University Hospital
ORs: odds ratios
PSA: prostate-specific antigen
