# Supplementary material for: Hair dye use, regular exercise, and the risk and prognosis of prostate cancer: multicenter case–control and case-only studies
Source: BMC Cancer. 2016 Mar 21;16:242. doi: 10.1186/s12885-016-2280-7 (PMC4800765; doi:10.1186/s12885-016-2280-7)
Supplement: Additional file 1: — The supplementary tables and figures. (DOC 418 kb) [file 12885_2016_2280_MOESM1_ESM.doc]

**Supplementary Figure Legends**

**Supplemental Fig. 1.**Kaplan-Meier survival curve of time stratified by clinical stages, Gleason score, and the PSA level: (**A)**. By clinical stages; (**B)**. By Gleason score; (**C)**. By the PSA level

**Supplemental Table 1.**Other demographic and clinical characteristics of 296 paired case-control study and 608 case-only study.

|  | Cases | | Controls | | |  | All cases  N = 608 | | |
| --- | --- | --- | --- | --- | --- | --- | --- | --- | --- |
| Variables | N = 296 | | N = 296 | | | P-value |
|  | N | (%) | N | | (%) |  | N | (%) | Death＃ (N=48) |
| Blood type* |  |  |  |  | |  |  |  |  |
| A | 68 | (23.0) | 56 | (18.9) | | 0.020* | 154 | (25.3) | 14 |
| B | 37 | (12.5) | 67 | (22.6) | |  | 118 | (19.4) | 11 |
| O | 103 | (34.8) | 100 | (33.8) | |  | 233 | (38.3) | 11 |
| AB | 16 | (5.4) | 13 | (4.4) | |  | 37 | (6.1) | 3 |
| Missing | 72 |  | 60 |  | |  | 66 |  | 9 |
|  |  |  |  |  | |  |  |  |  |
| Multivitamins |  |  |  |  | |  |  |  |  |
| No | 216 | (73.0) | 229 | (77.4) | | 0.1788 | 320 | (52.6) | 28 |
| Yes | 29 | (9.8) | 32 | (10.8) | |  | 196 | (32.2) | 12 |
| Missing | 51 |  | 35 |  | |  | 92 |  |  |
|  |  |  |  |  | |  |  |  |  |
| Tea consumption |  |  |  |  | |  |  |  |  |
| No | 148 | (50.0) | 163 | (55.1) | | 0.633 | 305 | (50.2) | 26 |
| Yes | 100 | (33.8) | 101 | (34.1 | |  | 272 | (44.7) | 22 |
| Missing | 48 |  | 32 |  | |  | 31 |  |  |
|  |  |  |  |  | |  |  |  |  |
| Coffee consumption |  |  |  |  | |  |  |  |  |
| No | 224 | (75.7 | 236 | (79.7) | | 0.854 | 486 | (79.9) | 42 |
| Yes | 28 | (9.5) | 28 | (9.5) | |  | 93 | (15.3) | 6 |
| Missing | 44 |  | 32 |  | |  | 29 |  |  |
|  |  |  |  |  | |  |  |  |  |
| Milk consumption |  |  |  |  | |  |  |  |  |
| No | 151 | (51.0) | 148 | (50.0) | | 0.771 | 302 | (49.7) | 30 |
| Yes | 140 | (47.3) | 144 | (48.6) | |  | 298 | (49.0) | 18 |
| Missing | 5 |  | 4 |  | |  | 8 |  |  |
|  |  |  |  |  | |  |  |  |  |
| Soybean milk consumption |  |  |  |  | |  |  |  |  |
| No | 222 | (75.0) | 233 | (78.7) | | 0.267 | 448 | (73.7) | 42 |
| Yes | 68 | (23.0) | 57 | (19.3) | |  | 152 | (25.0) | 6 |
| Missing | 6 |  | 6 |  | |  | 8 |  |  |
|  |  |  |  |  | |  |  |  |  |
| Instant noodle consumption |  |  |  |  | |  |  |  |  |
| No | 261 | (88.2) | 271 | (91.6) | | 0.340 | 555 | (91.3 | 41 |
| Yes | 27 | (9.1) | 21 | (7.1) | |  | 42 | (6.9) | 6 |
| Missing | 8 |  | 4 |  | |  | 11 |  |  |

* P-value < 0.05

＃prostate cancer specific death

**Supplemental Table 2.** Odds ratio (OR) for cases and controls according to hair dyes use and regular exercise. (Without missing data)

|  | Cases | | Controls | | Crude OR (95% CI) | | | | AOR (95% CI)^a^ | | | | AOR (95% CI)^b^ | | | |
| --- | --- | --- | --- | --- | --- | --- | --- | --- | --- | --- | --- | --- | --- | --- | --- | --- |
| Variables | N = 166 | | N = 196 | |
|  | N | (%) | N | (%) |
| Hair dyes |  |  |  |  |  |  |  |  |  |  |  |  |  |  |  |  |
| No | 119 | (71.7) | 159 | (81.1) | 1.00 |  |  |  | 1.00 |  |  |  | 1.00 |  |  |  |
| Yes | 47 | (28.3) | 37 | (18.9) | 1.70 | (1.04 | – | 2.79)* | 1.75 | (1.07 | – | 2.88)* | 1.87 | (1.09 | – | 3.24)* |
|  |  |  |  |  |  |  |  |  |  |  |  |  |  |  |  |  |
| Age of first use (yrs) |  |  |  |  |  |  |  |  |  |  |  |  |  |  |  |  |
| Never | 119 | (71.7) | 159 | (81.1) | 1.00 |  |  |  | 1.00 |  |  |  | 1.00 |  |  |  |
| ≥ 60 | 13 | (7.9) | 15 | (7.7) | 1.16 | (0.52 | – | 2.53) | 1.18 | (0.53 | – | 2.58) | 1.38 | (0.59 | – | 3.23) |
| 50-< 60 | 17 | (10.2) | 10 | (5.1) | 2.27 | (1.02 | – | 5.32)* | 2.39 | (1.06 | – | 5.63)* | 2.26 | (0.96 | – | 5.56) |
| < 50 | 17 | (10.2) | 12 | (6.1) | 1.89 | (0.88 | – | 4.21) | 1.95 | (0.90 | – | 4.35) | 2.11 | (0.91 | – | 5.06) |
| P for trend |  |  |  |  | 0.023 |  |  |  | 0.017 |  |  |  | 0.019 |  |  |  |
|  |  |  |  |  |  |  |  |  |  |  |  |  |  |  |  |  |
| Years of use (years) |  |  |  |  |  |  |  |  |  |  |  |  |  |  |  |  |
| Never | 119 | (71.7) | 159 | (81.1) | 1.00 |  |  |  | 1.00 |  |  |  | 1.00 |  |  |  |
| ≤ 10 | 22 | (13.2) | 22 | (11.2) | 1.34 | (0.70 | – | 2.54) | 1.37 | (0.72 | – | 2.62) | 1.43 | (0.72 | – | 2.86) |
| > 10 | 25 | (15.1) | 15 | (7.7) | 2.23 | (1.14 | – | 4.50)* | 2.30 | (1.17 | – | 4.67)* | 2.59 | (1.23 | – | 5.66)* |
| P for trend |  |  |  |  | 0.018 |  |  |  | 0.014 |  |  |  | 0.011 |  |  |  |
|  |  |  |  |  |  |  |  |  |  |  |  |  |  |  |  |  |
| Frequency of use (times per year) |  |  |  |  |  |  |  |  |  |  |  |  |  |  |  |  |
| Never | 119 | (71.7) | 159 | (81.1) | 1.00 |  |  |  | 1.00 |  |  |  | 1.00 |  |  |  |
| ≤ 6 | 24 | (14.5) | 22 | (11.2) | 1.46 | (0.78 | – | 2.74) | 1.48 | (0.79 | – | 2.79) | 1.52 | (0.78 | – | 3.01) |
| > 6 | 23 | (13.8) | 15 | (7.7) | 2.05 | (1.03 | – | 4.17) * | 2.16 | (1.08 | – | 4.40)* | 2.46 | (1.15 | – | 5.40)* |
| P for trend |  |  |  |  | 0.026 |  |  |  | 0.018 |  |  |  | 0.014 |  |  |  |
|  |  |  |  |  |  |  |  |  |  |  |  |  |  |  |  |  |
| Year of first use |  |  |  |  |  |  |  |  |  |  |  |  |  |  |  |  |
| No | 119 | (71.7) | 159 | (81.1) | 1.00 |  |  |  | 1.00 |  |  |  | 1.00 |  |  |  |
| After 1980 | 1 | (0.6) | 2 | (1.0) | 0.67 | (0.03 | – | 7.05) | 0.69 | (0.03 | – | 7.31) | 0.73 | (0.03 | – | 8.59) |
| Before 1980 | 46 | (27.7) | 35 | (17.9) | 1.76 | (1.07 | – | 2.91)* | 1.81 | (1.10 | – | 3.01)* | 1.94 | (1.12 | – | 3.38)* |
|  |  |  |  |  |  |  |  |  |  |  |  |  |  |  |  |  |
| Regular exercise |  |  |  |  |  |  |  |  |  |  |  |  |  |  |  |  |
| No | 70 | (42.2) | 85 | (43.4) | 1.00 |  |  |  | 1.00 |  |  |  | 1.00 |  |  |  |
| Yes | 96 | (57.8) | 111 | (56.6) | 1.05 | (0.69 | – | 1.60) | 1.07 | (0.70 | – | 1.64) | 1.07 | (0.66 | – | 1.72) |
|  |  |  |  |  |  |  |  |  |  |  |  |  |  |  |  |  |
| Frequency of exercise (times per week) |  |  |  |  |  |  |  |  |  |  |  |  |  |  |  |  |
| No | 70 | (42.2) | 85 | (43.4) | 1.00 |  |  |  | 1.00 |  |  |  | 1.00 |  |  |  |
| 1-6 | 35 | (21.1) | 40 | (20.4) | 1.06 | (0.61 | – | 1.85) | 1.11 | (0.63 | – | 1.94) | 1.11 | (0.60 | – | 2.04) |
| ≥ 7 | 61 | (36.7) | 71 | (36.2) | 1.04 | (0.65 | – | 1.66) | 1.05 | (0.65 | – | 1.69) | 1.04 | (0.62 | – | 1.77) |

Abbreviation: AOR: adjusted OR; OR: odds ratio; PC: prostate cancer.

a: Adjusting for age and family history of PC,

b:Adjusting for age, marital status, blood type, education, family history of PC, cigarette smoking, alcohol drinking and betel nut chewing

* P-value < 0.05.

**Supplemental Table 3.** Odds ratio for clinical stage of prostate cancer cases according to hair dyes use and regular exercise.

|  | Clinical Stage^◎^ | | | | | | |
| --- | --- | --- | --- | --- | --- | --- | --- |
| Variables | Localized | Locally Advanced |  |  | Bone Metastasis |  |  |
|  | N = 290 | N = 158 | Locally Advanced vs. Localized | | N = 154 | Bone Metastasis vs. Localized | |
|  | N (%) | N (%) | OR (95% CI) | AOR (95% CI)^a^ | N (%) | OR (95% CI) | AOR (95% CI) ^a^ |
| Hair dyes |  |  |  |  |  |  |  |
| No | 213 (73.4) | 105 (66.5) | 1.00 | 1.00 | 122 (79.2) | 1.00 | 1.00 |
| Yes | 75 (25.9) | 52 (32.9) | 1.41 (0.92-2.15) | 1.42 (0.86-2.34) | 31 (20.1) | 0.72 (0.44-1.15) | 0.75 (0.40-1.36) |
| Missing | 2 | 1 |  |  | 1 |  |  |
| Years of use |  |  |  |  |  |  |  |
| Never | 213 (73.4) | 105 (66.5) | 1.00 | 1.00 | 122 (79.2) | 1.00 | 1.00 |
| ≤ 10 years | 43 (14.8) | 23 (14.6) | 1.09 (0.61-1.88) | 1.21 (0.61-2.34) | 17 (11.0) | 0.69 (0.37-1.24) | 0.92 (0.42-1.95) |
| > 10 years | 28 (9.7) | 22 (13.9) | 1.59 (0.86-2.91) | 1.52 (0.75-3.08) | 13 (8.4) | 0.81 (0.39-1.59) | 0.69 (0.26-1.65) |
| Missing | 6 | 8 |  |  | 2 |  |  |
| Times of year |  |  |  |  |  |  |  |
| Never | 213 (73.4) | 105 (66.5) | 1.00 | 1.00 | 122 (79.2) | 1.00 | 1.00 |
| ≤ 6 times/yr | 39 (13.4) | 26 (16.5) | 1.35 (0.78-2.33) | 1.69 (0.88-3.22) | 15 (9.7) | 0.67 (0.35-1.24) | 0.94 (0.40-2.05) |
| > 6 times/yr | 29 (10.0) | 20 (12.7) | 1.40 (0.75-2.58) | 1.41 (0.67-2.90) | 14 (9.1) | 0.84 (0.42-1.63) | 0.80 (0.32-1.87) |
| Missing | 9 | 7 |  |  | 3 |  |  |

^a^ Adjusted age, marital status, blood type, education, family history of PC, smoking, alcohol and betel chewing

^◎^Tumor, node, metastasis system staging by American Joint Committee on Cancer (1997): Localized, T1/T2 N0 M0; Locally advanced, T3/T4 N1 M0; Bone Metastasis, M1

**Supplemental Table 3.** Odds ratio for clinical stage of prostate cancer cases according to hair dyes use and regular exercise (cont.)

|  | Clinical Stage^◎^ | | | | | | |
| --- | --- | --- | --- | --- | --- | --- | --- |
| Variables | Localized | Locally Advanced |  |  | Bone Metastasis |  |  |
|  | N = 290 | N = 158 | Locally Advanced vs. Localized | | N = 154 | Bone Metastasis vs. Localized | |
|  | N (%) | N (%) | OR (95% CI) | AOR (95% CI)^a^ | N (%) | OR (95% CI) | AOR (95% CI) ^a^ |
| Regular exercise |  |  |  |  |  |  |  |
| No | 91 (31.4) | 77 (48.7) | 1.00 | 1.00 | 84 (54.5) | 1.00 | 1.00 |
| Yes | 199 (68.6) | 80 (50.6) | 0.48 (0.32-0.71)* | 0.51 (0.31-0.82)* | 68 (44.2) | 0.37 (0.25-0.55)* | 0.36 (0.21-0.61)* |
| Missing | 0 | 1 |  |  | 2 |  |  |
|  |  |  |  |  |  |  |  |
| Times of exercise |  |  |  |  |  |  |  |
| Never | 91 (31.4) | 77 (48.7) | 1.00 | 1.00 | 84 (54.5) | 1.00 | 1.00 |
| 1-6 times/week | 106 (36.6) | 26 (16.5) | 0.29 (0.17-0.49)* | 0.30 (0.16-0.55)* | 26 (16.9) | 0.27 (0.16-0.44)* | 0.32 (0.17-0.62)* |
| ≥ 1 time/day | 90 (31.0) | 54 (34.2) | 0.71 (0.45-1.11) | 0.76 (0.44-1.30) | 41 (26.6) | 0.49 (0.31-0.7)* | 0.40 (0.21-0.73)* |
| Missing | 3 | 1 |  |  | 3 |  |  |
|  |  |  |  |  |  |  |  |
| Gleason Score |  |  |  |  |  |  |  |
| ≤ 6 | 168 (57.9) | 51 (32.3) | 1.00 | 1.00 | 33 (21.4) | 1.00 | 1.00 |
| 7 | 82 (28.3) | 64 (40.5) | 2.57 (1.64-4.06)* | 2.66 (1.55-4.61)* | 43 (27.9) | 2.67 (1.58-4.54)* | 2.91 (1.47-5.87)* |
| 8-10 | 37 (12.8) | 41 (25.9) | 3.65 (2.13-6.32)* | 4.92 (2.56-9.65)* | 76 (49.4) | 10.46 (6.15-18.21)* | 14.85 (7.45-31.04)* |
| Missing | 3 | 2 |  |  | 2 |  |  |
|  |  |  |  |  |  |  |  |
| Preoperative PSA (ng/ml) |  |  |  |  |  |  |  |
| < 10 | 120 (41.4) | 22 (13.9) | 1.00 | 1.00 | 7 (4.5) | 1.00 | 1.00 |
| 10-< 20 | 96 (33.1) | 38 (24.1) | 2.16 (1.21-3.94)* | 2.37 (1.22-4.71)* | 7 (4.5) | 1.25 (0.41-3.77) | 1.50 (0.38-6.42) |
| ≥ 20 | 67 (23.1) | 94 (59.5) | 7.65 (4.47-13.54)* | 8.41 (4.48-16.45)* | 134 (87.0) | 34.29 (16.18-84.73)* | 55.19 (19.88-201.37)* |
| Missing | 7 | 4 |  |  | 6 |  |  |

^a^ Adjusted age, marital status, blood type, education, family history of PC, smoking, alcohol and betel chewing

^◎^Tumor, node, metastasis system staging by American Joint Committee on Cancer (1997): Localized, T1/T2 N0 M0; Locally advanced, T3/T4 N1 M0; Bone Metastasis, M1

**Supplemental Table 4.** Prostate-cancer-specific death according to hair dye use and regular exercise in a Cox regression model. (Among patients with T1~4/N0~1/M0 of TMN stage) ◎

| N | Total  448 | | Prostate-cancer-specific death 14 | HR | (95% CI) | AHR^a^ | (95% CI) |
| --- | --- | --- | --- | --- | --- | --- | --- |
| Variables | N (%) | | N |  |  |  |  |
| Hair dyes |  |  |  |  |  |  |  |
| No | 318 | (71.5) | 8 | 1.00 |  | 1.00 |  |
| Yes | 127 | (28.5) | 6 | 1.57 | (0.51-4.55) | 1.74 | (0.45-6.62) |
| Missing | 3 |  | 0 |  |  |  |  |
|  |  |  |  |  |  |  |  |
| Years of use |  |  |  |  |  |  |  |
| Never | 318 | (73.3) | 8 | 1.00 |  | 1.00 |  |
| ≤ 10 years | 66 | (15.2) | 4 | 1.85 | (0.49-5.92) | 4.87 | (0.97-26.0) |
| > 10 years | 50 | (11.5) | 0 | 1.85e-9 | (0.00-1.52) | 01.13e-9 | (0-1.47) |
| Missing | 14 |  | 2 |  |  |  |  |
|  |  |  |  |  |  |  |  |
| Frequency of use (times per year |  |  |  |  |  |  |  |
| Never | 318 | (73.6) | 8 | 1.00 |  | 1.00 |  |
| ≤ 6 | 65 | (15.1) | 2 | 1.16 | (0.17-4.63) | 1.42 | (0.20-6.96) |
| > 6 | 49 | (11.3) | 3 | 1.63 | (0.35-5.75) | 2.31 | (0.41-11.16) |
| Missing | 16 |  | 1 |  |  |  |  |
|  |  |  |  |  |  |  |  |
| Regular exercise |  |  |  |  |  |  |  |
| No | 168 | (37.6) | 8 | 1.00 |  | 1.00 |  |
| Yes | 279 | (62.4) | 6 | 0.43 | (0.14-1.24) | 0.34 | (0.09-1.13) |
| Missing | 1 |  | 0 |  |  |  |  |
|  |  |  |  |  |  |  |  |
| Frequency of exercise (times per week) |  |  |  |  |  |  |  |
| Never | 168 | (37.8) | 8 | 1.00 |  | 1.00 |  |
| 1-6 | 132 | (29.7) | 2 | 0.34 | (0.05-1.36) | 0.14 | (0.01-0.86)* |
| ≥ 7 | 144 | (32.4) | 4 | 0.51 | (0.14-1.62) | 0.51 | (0.12-1.82) |
| Missing | 4 |  | 0 |  |  |  |  |

Abbreviation: AHR: adjusted HR; HR: hazard ratio.

* P-value < 0.05

a:Adjusting for clinical stage, PSA, age, marital status, blood type, education, family history of PC, cigarette smoking, alcohol drinking, and betel nut chewing.

◎Tumor, node, metastasis system staging by American Joint Committee on Cancer (1997): Localized, T1/T2 N0 M0; Locally advanced, T3/T4 N1 M0; Bone Metastasis, M1

**Supplemental Table 5.** Prostate-cancer-specific death according to hair dye use and regular exercise in a Cox regression model. (Among patients with M1 of TMN stage ) ^◎^

| N | Total  153 | | Prostate-cancer-specific death 34 | HR | (95% CI) | AHR^a^ | (95% CI) |
| --- | --- | --- | --- | --- | --- | --- | --- |
| Variables | N (%) | | N |  |  |  |  |
| Hair dyes |  |  |  |  |  |  |  |
| No | 122 | (79.7) | 27 | 1.00 |  | 1.00 |  |
| Yes | 31 | (20.3) | 7 | 0.81 | (0.32-7.77) | 0.85 | (0.32-2.01) |
| Missing |  |  | 0 |  |  |  |  |
|  |  |  |  |  |  |  |  |
| Years of use |  |  |  |  |  |  |  |
| Never | 122 | (80.3) | 27 | 1.00 |  | 1.00 |  |
| ≤ 10 years | 17 | (11.2) | 5 | 1.36 | (0.46-3.28) | 1.10 | (0.35-2.90) |
| > 10 years | 13 | (8.6) | 2 | 0.46 | (0.07-1.54) | 0.52 | (0.08-1.99) |
| Missing | 1 |  | 0 |  |  |  |  |
|  |  |  |  |  |  |  |  |
| Frequency of use (times per year |  |  |  |  |  |  |  |
| Never | 122 | (80.8) | 27 | 1.00 |  | 1.00 |  |
| ≤ 6 | 15 | (9.9) | 3 | 0.72 | (0.17-2.05) | 0.78 | (0.18-2.46) |
| > 6 | 14 | (9.3) | 4 | 1.13 | (0.33-2.90) | 0.92 | (0.25-2.75) |
| Missing | 2 |  | 0 |  |  |  |  |
|  |  |  |  |  |  |  |  |
| Regular exercise |  |  |  |  |  |  |  |
| No | 84 | (55.3) | 26 | 1.00 |  | 1.00 |  |
| Yes | 68 | (44.7) | 8 | 0.32 | (0.13-0.67) | 0.22 | (0.08-0.53) |
| Missing | 1 |  | 0 |  |  |  |  |
|  |  |  |  |  |  |  |  |
| Frequency of exercise (times per week) |  |  |  |  |  |  |  |
| Never | 84 | (55.6) | 26 | 1.00 |  | 1.00 |  |
| 1-6 | 26 | (17.2) | 5 | 0.74 | (0.25-1.78) | 0.54 | (0.15-1.57) |
| ≥ 7 | 41 | (27.2) | 2 | 0.11 | (0.02-0.38) | 0.07 | (0.01-0.26) |
| Missing | 2 |  | 1 |  |  |  |  |

Abbreviation: AHR: adjusted HR; HR: hazard ratio.

* P-value < 0.05

a:Adjusting for PSA, age, marital status, blood type, education, family history of PC, cigarette smoking, alcohol drinking, and betel nut chewing.

^◎^Tumor, node, metastasis system staging by American Joint Committee on Cancer (1997): Localized, T1/T2 N0 M0; Locally advanced, T3/T4 N1 M0; Bone Metastasis, M1

**Supplemental Table 6.** Other-cause death by hair dye use and regular exercise in a Cox regression model.

| **N** | **Total**  **608** | | **Other-cause death**  **31** | **HR** | **(95% CI)** | **AHR^a^** | **(95% CI)** |
| --- | --- | --- | --- | --- | --- | --- | --- |
| **Variables** | **N (%)** | | **N** |  |  |  |  |
| **Hair dyes** |  |  |  |  |  |  |  |
| No | 443 | (72.9) | 21 | 1.00 |  | 1.00 |  |
| Yes | 161 | (54.4) | 10 | 1.13 | (0.51-2.35) | 1.93 | (0.66-5.42) |
| Missing | 4 |  | 0 |  |  |  |  |
|  |  |  |  |  |  |  |  |
| Years of use |  |  |  |  |  |  |  |
| Never | 443 | (72.9) | 21 | 1.00 |  | 1.00 |  |
| ≤ 10 years | 85 | (14.0) | 4 | 0.86 | (0.25-2.25) | 1.74 | (0.36-6.58) |
| > 10 years | 64 | (10.5) | 4 | 1.20 | (0.35-3.15) | 2.33 | (0.60-7.50) |
| Missing | 16 |  | 2 |  |  |  |  |
|  |  |  |  |  |  |  |  |
| Frequency of use (times per year |  |  |  |  |  |  |  |
| Never | 443 | (72.9) | 21 | 1.00 |  | 1.00 |  |
| ≤ 6 | 83 | (13.7) | 1 | 0.25 | (0.01-1.18) | 1.59 | (0-1.73) |
| > 6 | 63 | (10.4) | 6 | 1.59 | (0.58-3.74) | 2.68 | (0.85-7.74) |
| Missing | 19 |  | 3 |  |  |  |  |
|  |  |  |  |  |  |  |  |
| **Regular exercise** |  |  |  |  |  |  |  |
| No | 252 | (41.4) | 16 | 1.00 |  | 1.00 |  |
| Yes | 353 | (58.1) | 15 | 0.62 | (0.30-1.25) | 0.52 | (0.19-1.39) |
| Missing | 3 |  | 0 |  |  |  |  |
|  |  |  |  |  |  |  |  |
| Frequency of exercise (times per week) |  |  |  |  |  |  |  |
| Never | 252 | (41.4) | 16 | 1.00 |  | 1.00 |  |
| 1-6 | 161 | (26.5) | 4 | 0.43 | (0.12-1.18) | 0.21 | (0.01-1.27) |
| ≥ 7 | 188 | (30.9) | 11 | 0.75 | (0.34-1.61) | 0.66 | (0.23-1.79) |
| Missing | 7 |  | 0 |  |  |  |  |
|  |  |  |  |  |  |  |  |
| **Clinical stage^◎^** |  |  |  |  |  |  |  |
| Localized | 290 | (47.7) | 11 | 1.00 |  | 1.00 |  |
| Locally advanced | 158 | (26.0) | 7 | 1.24 | (0.45-3.15) | 1.04 | (0.25-4.03) |
| Bone metastasis | 154 | (25.3) | 12 | 2.65 | (1.15-6.15)* | 2.77 | (0.59-14.11) |
| Missing | 6 |  | 1 |  |  |  |  |

Abbreviation: AHR: adjusted HR; HR: hazard ratio.

* P-value < 0.05

^a^Adjusting for clinical stage, PSA, age, marital status, blood type, education, family history of PC, cigarette smoking, alcohol drinking, and betel nut chewing.

^◎^Tumor, node, metastasis system staging by American Joint Committee on Cancer (1997): Localized, T1/T2 N0 M0; Locally advanced, T3/T4 N1 M0; Bone Metastasis, M1

**Supplemental Table 7.** Sensitivity analysis of prostate-cancer-specific death by hair dye use and regular exercise in a Cox regression model.

| **N** | **Total**  **445** | | **Prostate-cancer-specific death**  **47** | **RR** | **(95% CI)** | **ARR^a^** | **(95% CI)** |
| --- | --- | --- | --- | --- | --- | --- | --- |
| **Variables** | **N (%)** | | **N** |  |  |  |  |
| **Hair dye** |  |  |  |  |  |  |  |
| No | 325 | (73.0) | 34 | 1.00 |  | 1.00 |  |
| Yes | 116 | (26.1) | 13 | 1.08 | (0.53-2.08) | 1.43 | (0.58-3.42) |
| Missing | 4 |  | 0 |  |  |  |  |
|  |  |  |  |  |  |  |  |
| Years of use |  |  |  |  |  |  |  |
| Never | 325 | (73.0) | 34 | 1.00 |  | 1.00 |  |
| ≤ 10 years | 53 | (11.9) | 9 | 1.45 | (0.62-3.09) | 2.67 | (0.93-7.58) |
| > 10 years | 43 | (9.7) | 2 | 0.40 | (0.06-1.37) | 0.28 | (0.04-1.35) |
| Missing | 13 |  | 2 |  |  |  |  |
|  |  |  |  |  |  |  |  |
| Times of year |  |  |  |  |  |  |  |
| Never | 325 | (73.0) | 34 | 1.00 |  | 1.00 |  |
| ≤ 6 times/yr | 55 | (12.4) | 5 | 0.86 | (0.28-2.12) | 1.13 | (0.31-3.57) |
| > 6 times/yr | 51 | (11.5) | 7 | 1.36 | (0.53-3.10) | 1.91 | (0.60-5.69) |
| Missing | 14 |  | 1 |  |  |  |  |
|  |  |  |  |  |  |  |  |
| **Regular exercise** |  |  |  |  |  |  |  |
| No | 188 | (42.3) | 33 | 1.00 |  | 1.00 |  |
| Yes | 255 | (57.3) | 14 | 0.27 | (0.14-0.52)* | 0.23 | (0.10-0.51)* |
| Missing | 2 |  | 0 |  |  |  |  |
|  |  |  |  |  |  |  |  |
| Times of exercise |  |  |  |  |  |  |  |
| Never | 188 | (42.3) | 33 | 1.00 |  | 1.00 |  |
| 1-6 times/week | 96 | (21.6) | 7 | 0.37 | (0.15-0.82)* | 0.66 | (0.22-1.82) |
| ≥ 1 time/day | 155 | (34.8) | 6 | 0.19 | (0.07-0.43)* | 0.44 | (0.07-0.65)* |
| Missing | 6 |  | 1 |  |  |  |  |
|  |  |  |  |  |  |  |  |
| **Clinical stage^◎^** |  |  |  |  |  |  |  |
| Localized | 194 | (43.6) | 7 | 1.00 |  | 1.00 |  |
| Locally Advanced | 124 | (27.9) | 7 | 1.60 | (0.53-4.78) | 0.74 | (0.18-2.72) |
| Bone Metastasis | 122 | (27.4) | 33 | 9.91 | (4.46-25.19)* | 8.18 | (2.58-29.15)* |
| Missing | 5 |  | 0 |  |  |  |  |

^◎^Tumor, node, metastasis system staging by American Joint Committee on Cancer (1997): Localized, T1/T2 N0 M0; Locally advanced, T3/T4 N1 M0; Bone Metastasis, M1.

* P-value < 0.05

**Supplemental Fig. 1:** Kaplan-Meier survival curve of time stratified by clinical stages, Gleason score, and the PSA level: (A). By clinical stages; (B). By Gleason score; (C). By the PSA level

1. **Kaplan-Meier survival curve by clinical stages**

1. **Kaplan-Meier survival curve by Gleason score**

1. **Kaplan-Meier survival curve by the PSA level**
